# Bibliographical Notices

**Published:** 1840-01-01

**Authors:** 


### Periscope;

OR,

### CIRCUMSPECTIVE REVIEW.

" Ore trahit quodcunque potest, atque addit acervo."

### Notices of some New Works.

Observations on the Disorder of the General Health of Females, called Chlorosis; shewing the true Cause to be entirely independent of peculiarities of sex. By *Samuel Fox*, Surgeon. pp. 132. London, S. Highley, Fleet-street.

Mr. Fox informs us, in his Preface, that:—

" His chief endeavour has been to direct the reader's attention to the fact that, the disorder called Chlorosis, is not peculiar to the Female Sex, and its occurrence limited to any particular period of life;—but that it is solely a complication of affections (entirely independent of sexual formation) resulting from, and almost invariably attending a peculiar functional derangement of the liver and digestive economy: and further, that the Chlorotic appearance as it is termed, so often to be observed, not only in young females during the earlier years of growth and long before the period when puberty commences, but in those also of adult age, whether married or single, as well as in the young and delicate of the male sex,—may be thus easily accounted for.

The Author sincerely hopes that, the simple mode of treatment, in accordance with his notions of the true cause of this disordered health (and from his long experience of its efficacy, he can confidently assert it to be by far the most successful,)—will induce those of his Medical Brethren who may adopt it, to throw aside the use of most, or all of those disgusting and too often dangerous medicines, called emmenagogues—which not only are constantly proving ineffectual for the object of their administration, but even tend in a great degree, to increase the real and only disease of the Chlorotic female."

We beg to observe that the capitals are Mr. Fox's own. Of course he best knows what is admirable and what is not; yet, on the principle that " good wine needs no bush," we think that both capitals and italics should be very sparingly used.

It appears that Mr. Fox was first induced to study Chlorosis with attention, from observing that youths and delicate males were, as well as females, subject to the disorder.

" From practical observation, I conceived the immediate source of the complaint in either sex, to be an obstruction in the capillary extremities of the biliary vessels, giving rise to chronic functional derangement of the liver. Acting on this belief, I discarded the idea of Chlorosis being an idiopathic, or primary disease,—and I adopted a mode of treatment in accordance with my view of the case, which, for upwards of thirty years, has never deceived me."

He goes on to observe:—

" Is not the retention of the menses the cause of the general ill-health of the female? I contend that it is not—on the contrary, that the retention of this natural discharge is only one of many other symptoms and sympathetic affections, whose common origin is derangement of the biliary apparatus of the liver; and I also contend, that, in by far the greater proportion of cases, this functional derangement of the liver is primary, or idiopathic; and that the symptoms and affections called dyspepsia, are only the results of the sympathy existing between the liver and the other chylopoietic viscera. At the same time, I admit that there are also many instances to be met with, where an idiopathic dyspepsia was clearly the cause of the functional derangement of the liver. Yet, this, in no wise, alters or impairs my position of the liver being the immediate or proximate cause of the retention or suppression of the menses; and I can confidently assert that, unless the remedies be such as possess a salutary influence over the liver, a healthy return or appearance of the catamenia can never be expected.

A little farther on Mr. Fox takes even bolder ground. He does not scruple to say:—

"From the view which I have taken, and the due consideration which I have given to the various opinions on the subject under consideration; and notwithstanding all that has been advanced in support of my opinion, still I am fully convinced, that it is a matter of not the slightest consequence, whether the menstrual discharge be a SECRETION or NOT; and however peculiar it may be considered in relation to its characters and qualities, I notwithstanding dissent from these medical authorities, still further on the subject of menstruation, for I do not consider it to be requisite for the health of the human female, that she should ever MENSTRUATE AT ALL. By this, I mean that the periodical loss of a certain quantity of blood or a fluid from the uterus, is not required for the safety of the general health, and that the catamenial discharge is merely a local relief, or resolution of the vessels, (as Dr. Locock very properly terms it) when they have become periodically and healthily congested."

From this it must be evident that Mr. Fox is no temporiser. His is no sickly, milk and water theory. He goes the whole hog against common opinion, and either he or it is quite right or wrong. Mr. Fox supports his position by the following argument:—

"If menstruation be necessary to the health of the female, as it is said to be by most medical writers, then it is absolutely necessary that all females should continue to menstruate during the whole period of life; no exception can be admissible—no female, according to this rule, can be without the function, and, at the same time, be healthy; for, if otherwise, the health would terminate at the middle period of life, when the cessation of the menses usually takes place; since she would then be deprived of that function which had been hitherto so essentially necessary for her health. Again, if menstruation is so important to the female during the early part of life, that is, from about the age of fourteen, at which it usually commences,—at a time, it must be remembered, when the constitutional faculties are in full vigour and energy,—and when one would think the female required no such aid to support her health.—How, then, does it happen that the function is no longer demanded for the security of her health at that period of her life, when the powers of her constitution are becoming not only debilitated, but, in fact, decayed; and when too she stands most in need of all the aid derivable from any source, or from the continuance of any function? This does not appear to me to be reasonable: for, menstruation is either important for the safety of the general health all through the course of life—or the health does not require it at any period of life."

There is this objection to such reasoning. Before the age of puberty and after the age of menstruation, the female constitution is different from what it is during the menstruating age. Prior to it the uterine system is not evolved—subsequent to it that system atrophies—and it may be reasonably thought that an evacuation requisite while it is subject to congestion is not requisite when it is not subject to congestion. Mr. Fox may reply that this favours the idea of such evacuation being needed for the uterus, but not for the health. The uterine system, however, is so linked with the general, during the period of fertility, that what affects the one will affect the other. And, after all, the question resolves itself into one of fact. If, (a female being in good health,) a cause acts directly on the uterus and disturbs its function, and if deranged health succeeds, that proves, so far as any thing in medicine is proved, that uterine disturbance breeds general. And who has not witnessed such a case?

Mr. Fox carries on the argument, or puts it in another shape:

"It is also true that, if the female is deprived of the ovaria, she is no longer capable of becoming impregnated, and she no longer menstruates. So also is it, with regard to the male sex. The testes are the agents for producing the fecundating fluid, consequently, a deprivation of them, renders him unable to impregnate the female. But, does the health of either party decline in consequence of their losing the faculty of pro-creating their species? The healthy eunuch is an answer as regards the male; and so far from the discontinuance of the monthly discharge in the female being prejudicial to her, it is even found that the health of those individuals, in whom the deprivation of the ovaria has been either congenital or chirurgical, is usually of the best. And, we know that animals of both sexes, under similar circumstances, thrive in a far greater proportion, than those possessed of the means of propagation."

But if that which, some way or another, produces the congestion, be absent, and consequently no congestion takes place, then the relief of the congestion may be well supposed to be unnecessary. This applies to either testes or ovaria. Persons may get on well enough without them, though it does not, therefore, follow that, while they are present, their functions can be interfered with harmlessly.

Mr. Fox, indeed, may be said to give up the point, for he states :—

"It appears to me that, the real cause of the disordered state of the system which so frequently supervenes, the sudden cessation of the menstrual discharge from cold or fright for example, is the already congested vessels of the uterus becoming rapidly engorged and producing, by that means, sympathetic nervous irritation throughout the whole system, and thus effecting a temporary change in the general health, in accordance to a well established law in pathology, that no local excitement or irritation can continue long without producing an effect on the system at large. A similar effect will be produced in either of the sexes, by a sudden morbid engorgement of the vessels of any of the organs of the body, as we know by daily experience; nothing is more common than a congestion of the vessels of the mucous membrane of the nose, from taking cold suddenly, and, in the first instance, the secretion is always suspended."

We will not say much of the reasoning or of the analogies here. They are not very clear to our understanding. But the essential point is conceded, that general derangement succeeds suppression of the catamenia; and surely this would be likely to succeed whether that suppression resulted from external or internal causes. The quo modo of the general derangement is a matter of speculation—the fact is the main thing.

We fear that Mr. Fox is either a very hasty reasoner or else that he lacks experience. Take the following passage :—

"It has been recently asserted, that symptoms resembling GONORRHŒA have been the consequences of sexual intercourse during the period the woman was menstruating. Now, we know very well, that any cause of excessive irritation may produce inflammation in either the serous or mucous membranes. Thus, the serous membranes will readily take on the adhesive inflammation, whilst those of the mucous membranes will, on the contrary, take on the suppurative inflammation; it may follow, therefore, that an over-anxious and excited coïtus may produce, in the male, an increased action in the urethra to an extent, sufficient to bring on the suppurative inflammation of its mucous membrane, and hence a diseased secretion from the lacunae glands, simulating virulent gonorrhœa. It may be fairly presumed, there must have been, in the cases alluded to, an over-appetence for coïtion on the part of the male, to have induced him to associate with a female under her circumstances. But the idea that the menstrual discharge is in any way noxious, and particularly that it possesses INFECTIOUS PROPERTIES capable of producing gonorrhœa, is totally at variance with medical experience. Moreover, if EVERY FEMALE is PERIODICALLY INFECTED with LOCAL DISEASE because she MENSTRUATES, then the very many persons who have daily intercourse with females who are menstruating, will, as a matter of course, become INFECTED with GONORRHŒA; but we know that such is not the fact; for there are few men who have so complete a control over their passions as to forego the gratifications they anticipate, merely on that account; and the menstrual discharge must infect all or none; there can be no qualification."

*First,* Mr. Fox denies by implication what most men of experience will affirm —*that* urethral discharge undistinguishable from gonorrhœa in the male, will follow connection with a menstruating female;—*secondly,* Mr. Fox attributes such discharge to particular excitement in the male, though, in the majority of cases of this sort, the fact of menstruation was not known at the time of coition;—*thirdly,* Mr. Fox libels men by saying, that few would fail to gratify their passions with a menstruating woman, whereas we believe the fact to be, that few so gratify them;—*thirdly,* he asserts that if a discharge be capable of exciting disease it must always when applied excite disease, the fact being notoriously the contrary: two men may have connection with a clapped woman, and while one gets the clap the other does not. A certain condition of the recipient as well as of the giver is requisite.

Passing to the predisposing causes of chlorosis, we think Mr. Fox's observations judicious:—

"Females in almost all parts of the world are chiefly confined to the domestic management of a family, and it would seem that nature, in her wisdom, fitted and intended them for such employments; seeing that the delicacy of their frames and constitutions, is inadequate to follow the more powerful and laborious avocations. It is, however, much to be regretted that such confined occupations, although necessarily required, are not the most beneficial to the health of the individuals engaged in them, who are thus deprived of exercise in the open air; for nothing tends to disorder the functions of the body so much as close confinement within doors, which, if persisted in, for any length of time, is sure to lead to this derangement of the general health of which we are now speaking. Unhappily, it is too often the case that very many females, from their slender circumstances in life, are urged by necessity to support themselves at some sedentary employment, and are thus deprived, day after day, of all means of exercising the body, by which only a free circulation of the animal fluids can be maintained. Hence, inactivity of the bowels and obstinate costiveness ensue, occasioning morbid irritation throughout the whole course of the intestines, which soon gives origin to functional, and, if not seasonably corrected, to organic disease of the liver, from which springs a host of affections destructive of the general health. If we add to this the smoke and contamination of a large metropolis or crowded towns, the constant inhalation of miasmatic vapour always existing in such places, the frequent atmospheric changes of our climate and the exposure of the external surfaces of the body to these vicissitudes, the irregular mode of living, and various moral causes, we cannot wonder greatly that the health of the weak and delicate female should so soon become deranged."

That mauvaise honte of young females which leads them so sadly to neglect themselves is indeed much to be lamented.

How chlorosis results from obstructed pores of the liver, Mr. Fox sets forth in petto in this paragraph:—

"It appears therefore manifest, from the foregoing observations, that under a deterioration of the hepatic function, an unhealthy condition of the stomach and chylopoietic viscera may be produced, the glandular system greatly obstructed, the secretions and excretions unhealthily performed, an impoverished condition of blood and juices generally obtains, and a consequent loss of tone and energy throughout the whole course of circulation result; thus, also, the vessels generally throughout the system, are insufficiently supplied with blood, and consequently, those of the uterus amongst the rest, which occasions the absence of the menses at the expected period,—general ill-health prevails,—and this is what AUTHORS call CHLOROSIS."

We must candidly confess that, on attentively perusing the work before us, the exact mode in which Mr. Fox proves his case is a mystery to us. Certainly morbid anatomy furnishes none of the proofs, nor is the precise train of reasoning very palpable. He observes indeed :—

"It has been shown by Dr. Johnson and others, that there may be considerable and long-enduring functional disturbance of stomach and intestines, without its being possible, after death, to discover any structural disease of those organs, or at least to such an extent, as to warrant our assuming it as the sole or chief cause of the derangement during life; it is therefore fair, similarly to conclude that there may be a long continued deranged function of liver, without THAT VISCUS being in a perceptible state of organic disease; and this I believe to be the fact in the complaint called Chlorosis; though it is obvious that continued functional derangement of any organ, must ultimately produce structural disease."

So morbid anatomy may be dispensed with. But, after a fund of reasoning, Mr. Fox winds up :—

"This then, is the only rational view of the cause of CHLOROSIS in LOVE-SICK MAIDS; viz., that by long-continued grief or disappointment, the functions of the stomach and liver have become deranged; the secretion of a due quantity of bile ceases, the digestion and chylification are greatly impaired, and from a deficiency, or alteration in the properties of the gastric juice, the appetite fails, or is rendered capricious; and, as little or no good blood is made, the cheeks, gums, and lips, lose their fine red colour, whilst the long train of symptoms and affections, already noticed, follows. BUT AUTHORS CALL DISAPPOINTMENT IN THE OBJECT OF DESIRE—LOVE-SICKNESS—A CAUSE OF CHLOROSIS; but, on this subject I shall somewhat enlarge, when noticing some of the remedies that have been recommended for the removal of Chlorosis."

Mr. Fox arrives after several chapters at the treatment of chlorosis. He is indignant at the idea of matrimony.

"It is well known, he says, that the general appearance of the female under this disordered state, is any thing but attractive and prepossessing; on the contrary, she is rather disgusting to the opposite sex, the face having lost its vivid color, and become pale, or of a yellowish hue, and the whole body flaccid, with œdematous swellings. How is it therefore, to be expected, under all these disadvantageous circumstances, that the remedy here recommended can, by any possibility, be within reach of the female, even supposing she were so inclined; and were it actually at the risk of character and of all moral feling, to be resorted to, in the way recommended, it would nevertheless fail to produce the effect, the author is led to expect. It is well known that married women, and those who are given to immoral indulgencies, are equally liable to this disordered state of the general health, called Chlorosis; medical practitioners of observation, find this frequently to be the fact; indeed, it is almost daily occurring. Many remarks are made when a young woman is existing under this complication of affections of disordered health, THAT MATRIMONY IS THE ONLY CURE: the absurdity, and and I must say, obscenity of this observation, in my opinion, displays a want of information, or rather a total ignorance of the real nature of the disease in existence."

We confess that the fact of married females being *equally* liable with the unmarried to chlorosis, startles us. It is quite new; and, obscene as the idea may be, we certainly have seen matrimony prove "a sovereign medicine" after the Pharmacopœia had done its best, or worst, in vain.

Passing over various criticisms on various remedies, good, bad, and indifferent, we present our author's own plan of treatment.

"The medical treatment must consist chiefly in arousing the liver to a more active and healthy performance of its offices; the bowels are in the first instance to be thoroughly evacuated of their offending contents, and they are to be afterwards kept from any accumulation, by the occasional use of some aperient medicine; but not in a powerful degree. Infusion of senna, Epsom salts and senna, or rhubarb either in substance or infusion, will be found generally to answer the purpose; of course, selecting such as are found by experience to be best adapted to each individual case. The bowels having been brought under proper command, mercury should be judiciously administered, until a more healthy secretion of bile shall be obtained; in fact, the healthy functions of the liver must be restored, or very little advantage will result from any, however elaborate mode of treatment. Other means must also be had recourse to, to restore a healthy condition of the system generally. Dr. Marshall Hall recommends sponging the body with salt and water, and afterwards using friction for some time with a coarse towel;—I have adopted this plan in many instances, and have found it exceedingly useful—it may, therefore, be amongst the other arrangements, as also the free application of the flesh-brush. By these means, a determination of the blood to the surface will be promoted, a matter of considerable importance; pure and frequent change of air, with moderate exercise, when the patient can bear the fatigue, will also be of great advantage in inducing the same object."

We must say that this smacks a little of the mountain in labour. The treatment here set down is just what five doctors out of six would pursue. And we cannot but remark on the sanguine temperament of Mr. Fox, *or* on his wonderful good fortune, when he has found habitual costiveness almost invariably cured by brown bread. Who of us has not prescribed brown bread for costiveness? And who has not seen it of some service? but who, save Mr. Fox, has had the luck of finding it almost invariably successful?

In conclusion, we would hint that Mr. Fox has given no proofs of this exclusive theory of Chlorosis—that he has not shewn that the uterine system is never primarily at fault—that he has not proved that the suppression of the catamenia is of no consequence—and that he has not convinced us that emmenagogues are always useless or necessarily dangerous.

Every body knows that chlorosis is frequently the result, rather than the cause of deranged general health. But we should be sorry to go so far as to assert that the uterine system *never* takes the initiative in the disease. The great general facts are always the truest; and when we see that young unmarried women compose the vast majority of the subjects of chlorosis, and when we reflect on the constrained and artificial state in which their uterine system is kept by the usages of society, we cannot but suspect that that system plays the most important part in the phenomena of the disease.

At the same time the old and the pernicious notions respecting the treatment of chlorosis, are fast wearing away, if they have not quite disappeared from the minds of intelligent practitioners. Emmenagogues are no longer looked on as specifics, but the uterine system is appealed to through the medium of the general health, both physical and mental. Yet it would be folly to do away with emmenagogues altogether, and experience has shewn that some remedies of that description may be usefully combined with such as enjoy a more extended operation. Such is the common sense view and, we believe, the true one, of the treatment of chlorosis.

